# Prevalence and first molecular identification of *Sarcocystis* species in feces of domestic dogs (*Canis familiaris*) in Egypt

**DOI:** 10.1186/s12917-023-03841-8

**Published:** 2023-12-18

**Authors:** Ismail S. Elshahawy, Marwa Fawaz, Aya Gomaa, Eman Mohammed

**Affiliations:** Department of Parasitology, Faculty of Veterinary Medicine, South Valley University, Qena, 83523 Egypt

**Keywords:** Domestic dogs, *Sarcocystis tenella*, PCR, Faeces, Molecular identification

## Abstract

**Background:**

*Sarcocystis* species are obligatorily heteroxenous protozoan parasites with predator–prey life cycles. Global Knowledge about the epidemiology and the distribution pattern of different *Sarcocystis* species in dog feces are very scarce. Therefore, the current investigation was conducted to declare the occurrence of *Sarcocystis* in the fecal specimens of the most common canids in Egypt, the domestic dogs, and to identify the species present using various parasitological and molecular approaches.

**Methods:**

A total of 100 dog fecal samples were collected and screened using fecal sugar flotation test for the presence of *Sarcocystis* oocysts/sporocysts. Additionally, thirty samples were used for genomic DNA extraction. The 18S rRNA gene fragment was the target of primers for a PCR, followed by purification and sequencing of the amplicons.

**Results:**

Currently, the results obtained reviewed that 4% of fecal samples were positive for *Sarcocystis* spp. using LM. Additionally, *Sarcocystis* spp. were verified in sixteen dogs (53.3%, 16/30) using PCR and subsequent sequencing protocols. Statistically, insignificant difference in prevalence of sarcocystosis relative to age and gender was noticed. Morphologically, the detected sporocysts measured 13.2–16.0 × 9.4–11 μm. Based on the 18S rRNA gene, sequencing analysis of amplicons from sporocysts DNA revealed 99.82% nucleotide homology with published *S. tenella* partial nucleotide sequences from sheep in Iraq and Iran.

**Conclusions:**

This is the first molecular evidence in support of the final host role of domestic dogs in the life cycle of *S. tenella* in Egypt, which provides a precious diagnostic tool for further epidemiological studies and for the assessment of the effectiveness of control measures for this disease.

**Supplementary Information:**

The online version contains supplementary material available at 10.1186/s12917-023-03841-8.

## Introduction

*Sarcocystis* species are unique intracellular protozoan parasites with an obligatory two-host life cycle based on an intermediate-definitive host association. The definitive host contract the infection through oral uptake of the cysts containing infective bradyzoites within fresh or poorly cooked infected meat of herbivores livestock, which generate the sexual phases in their intestinal tract and terminate in the development of oocysts /sporocysts, which are then shed in the faeces [1, 2].

Canids are one of the most significant animal species that serve as a host and reservoir for various parasites of concern to the veterinary and public health. Canids can excrete larvae, oocysts and eggs of various enteric and respiratory parasites resulting in parasitic diseases with potentially severe complications [3, 4]. The domestic dog (*Canis familiaris*), in particular, is regarded as the most common predator host for several *Sarcocystis* species, including *Sarcocystis cruzi*, *S. levinei, S. tenella*, *S. arieticanis, S. cameli*, *S. capracanis*, *S. hircicanis* and *S. miescheriana* with broad range of herbivorous intermediate hosts including cattle, buffalo, sheep, camel, goat and pig [2, 5, 6]. Dogs serve also as intermediate hosts for *S. caninum*, and *S. svanai*, associated with canine clinical muscular sarcocystosis based on the published revisions of [7]. *Sarcocystis* spp. are scarcely pathogenic to the carnivores final host; however, acute to chronic diarrhea may occur. On the contrary, herbivorous intermediate host usually suffer from extensive tissue damage and consequent increased mortality with economic losses [8, 9].

*Sarcocystis* species have been classified and identified based on the morphological investigation of sarcocysts within tissues of the intermediate host by means of light and transmission electron microscopy, and through detection of their final hosts experimentally [10]. Likewise, the direct microscopic observation of sporulated oocysts and sporocysts in fresh fecal smears is considered as the conventional diagnostic tool for detection of *Sarcocystis* spp. infection in the definitive hosts. However, these stages from most *Sarcocystis* spp. are morphologically indistinguishable, contrastingly; to the other apicomplexan protozoa. Therefore, microscopy has no taxonomic role in *Sarcocystis* species, because sporocysts of various species are quite similar. Recently, most of *Sarcocystis* species have been categorized based on their molecular characters using appropriate DNA markers amplified in polymerase chain reactions (PCR) followed by comparisons of the obtained nucleotide sequences. This offers more reliable method to identify *Sarcocystis* spp. [11–13]. Knowledge on molecular characterization of various of *Sarcocystis* species came mostly from identification of muscle cysts in tissue of the intermediate hosts, and there is a scarcity on the genetic characters of *Sarcocystis* oocysts/sporocysts in feces of the definitive hosts Therefore, the current study was designed to determine the prevalence of *Sarcocystis* species from dog faeces in Aswan governorate, Upper Egypt employing various parasitological and molecular approaches.

## Materials and methods

### Animals and sampling

The present survey was conducted in Aswan governorate in the southern district of Egypt. This area is bounded by 24° 5′ 20.18″ N latitude and 32° 53′ 59.39″ E longitude. Furthermore, the study region is characterized by livestock farming, particularly bovine and ovine herds with agricultural events as a source of income. It is considered as a nice place for prevailing of stray dogs due to their warm climate throughout the year.

Throughout this survey, a total of 100 domestic dogs mostly lived as strays particularly around abattoirs, were screened for the occurrence of protozoan oocysts/sporocysts. Fecal samples from all screened dogs were taken and transferred to the Parasitology Lab., Faculty of Veterinary Medicine, South Valley University for parasitological analysis. Dog's age and gender were also considered.

### Microscopical investigations

The collected fresh fecal samples were assessed using fecal flotation technique for identification of *Sarcocystis* stages. In brief, three grams of faeces from each dog were well mixed with 16 ml of Sheather's sucrose solution (454 g granulated sugar; 355 ml tap water and 6 ml formaldehyde 37%; specific gravity = 1.27 [14]. The above mentioned mixture was strained through a fine sieve (0.6 mm mesh) by continuous and thorough mixing with a glass rod. The suspension was transferred to a 15-ml centrifuge tube and centrifuged at 2000 g for 3 min. With the help of a glass rod, a drop of the flotation solution was transferred to a microscopic slide by touching the surface of the solution in the centrifuged tubes. The microscopic slide was examined under 100 × magnification for existence of *Sarcocystis* oocysts/sporocysts using an Olympus optical microscope as per the outlined schemes done by Dryden et al. [15].

### Molecular and phenotypic analysis

#### Genomic DNA isolation

Out of the 100 samples screened, 30 samples were selected for molecular testing, and the genomic DNA was extracted by using a stool DNA Kit, D4015-01, Omega Bio-TEK, USA) according to the manufacturer’s instructions. The isolated DNA was stored at – 20 °C until the PCR analysis.

#### PCR amplification

Oligonucleotide primers of Sar1 genes were supplied from Macrogen (Korea) and their sequences were illustrated by Bahari et al. [16] as follow Sar-F1forward 5'GCACTTGATGAATTCTGGCA3' and Sar-R1 reverse 5' CACCACCCATAGAATCAAG 3') for amplification of 18S ribosomal DNA genes. Briefly, reaction mixture for PCR contained 50 ng of genomic DNA from stool sample, 50 pmol each of the primers, 200, uM each of dNTPs, 5uL of 10 X PCR buffer (containing 100 mM Tris-HCI at pH 9, IS mM MgCI 2, 500 mM KCI), and 1.0 U Taq DNA polymerase (Promega) in a final volume of 50 μl. Thermal cycling conditions were as follow, pre-denaturation step at 94° C for 5 min and 30 cycles of denaturation at 94° C for 45 s, annealing at 55 °C for 1 min and extension at 72° C for 1 min with a final elongation step of 7 min at 72 °C at the last cycle [17].

### Visualization of the amplified PCR Products

Electrophoreses of PCR product was applied on 1.5% agarose gel (Agarose, universal, peq GOLD, peqlab Germany) in 1 × TBE buffer. About 20 μl of the PCR products was loaded into the gel. Gene marker 100 bp DNA ladder (peqGOLD 2 kb DNA-Ladder, Peqlab, VWR was used to specify the amplicons' size, stained with Ethidium Bromide (0.5 μg/ml) and visualized under the UV light. A gel documentation system (DigiDoc-It® Imaging System (UVP, UK) was used for recoding the result and analyzed through Totallab software).

### Genomic DNA Sequencing and phylogenetic relationship

PCR products were purified using E.Z.N.A. Gel Extraction Kit (Omega Bio-TEK, USA). The sequence reaction was carried out by Macrogen Inc., Korea and the purification process was conducted utilizing Micro spin column. The obtained DNA sequences by the ABI PRISM 3100 Genetic Analyzer (Micron-Corp. Korea) were exposed to BLAST search for sequence identity using the NCBI-BLAST website (https://blast.ncbi.nlm. nih. gov/Blast.cgi) according to methods described by Altschul et al. [18]. The obtained nucleotide sequences were deposited in the GenBank under the accession number ON421649. Maximum Likelihood, Neighbor Joining, and Maximum Parsimony methods were used to construct the phylogenetic trees based on 18 s rRNA sequences in MEGA7 [19].

### Statistical analysis

The statistical analysis of the variation of *Sarcocystis* spp. prevalence in dogs, in relation to the screened animal and epidemiological data, was performed with chi-square (*χ*2) test, using IBM SPSS Statistics for Windows, Version 21.0 (IBM Corp., Armonk, NY, USA). A *p* value ≤ 0.05 was considered significant according to Serra-Freire [20].

## Results

### Prevalence of Sarcocystis spp. in feces of screened dogs from Egypt

*Sarcocystis* stages were found in 4 (4%) out of 100 screened dogs using the coproscopic analysis, whereas sixteen dogs (53.3%) were found to be positive for *Sarcocystis* 18S rRNA by PCR approach. The statistical significance of this noticeable difference was determined between both diagnostic approaches (χ2 = 21.572, *P* =  < 0.0001) (Table 1). Also, the same table declared that old aged dogs (21.95%) were more disposed to *Sarcocystis* infection as compared to younger age category (4.2%). However, insignificant difference was observed between the different age categories (χ2 = 3.678, *P* = 0.05). Correspondingly, the prevalence of *Sarcocystis* infection was higher among the male dogs (19.05%) as compared to the female counterpart (8.7%), with an insignificant association (χ2 = 1.223*, P* > 0.05) between the prevalence rates.

**Table 1:** Occurrence percentages of *Sarcocystis* infection relative to age and gender of dogs by coprological and PCR analysis

Variables		Coproscopy		PCR on genomic DNA extracted directly from feces		Total	
		**Examined**	**Infected (%)**	**Examined**	**Infected (%)**	**Examined**	**Infected (%)**
**Sex**	**Male**	**66**	**4 (6.06)**	**18**	**12 (66.66)**	**84**	**16(19.05)**
	**Female**	**34**	**0 (0)**	**12**	**4 (33.33)**	**46**	**4(8.7)**
**Age**	** < 2 years**	**38**	**0 (0)**	**10**	**2(20)**	**48**	**2(4.2)**
	** ≥ 2 years**	**62**	**4 (6.45)**	**20**	**14(70)**	**82**	**18(21.95)**
**Total**		**100**	**4 (4)**	**30**	**16 (53.33)**	**100**	**20(20)**
***P***		** < 0.0001**					

Generally, as compared with coprological analysis, the detection rate of *Sarcocystis* spp. was significantly higher (χ2 = 5.4, *p* = 0.020, r = 0.6, Fisher's Zr = 0.693, 95% C.I. 0.1085–1.277) when a molecular method was employed.

The current results publicized that the PCR technique is a sensible scheme for inspection of *Sarcocystis* stages in dogs as compared to the coproscopical tool. Moreover, coproscopic tool recognized 4 confirmed positive samples out of the 16 specimens documented by PCR, yielding a sensitivity of 25%. However, out of the 14 samples verified as negative by PCR, 2 cases were revealed positive by coproscopy, giving a specificity of 85.7%. The relationship between coproscopy and PCR was slight with Kappa agreement test = 0.103 (Table 2).

**Table 2:** Diagnostic performance of coproscopical analysis and PCR tool for detecting *Sarcocystis* stages in dog feces

**Coproscopy**	**PCR**		**Sensitivity** **(95% C.I.)**	**Specificity** **(95% C.I.)**	**PPV** **(95% C.I.)**	**NNP** **(95% C.I.)**	^**K**^ values **(95% C.I.)**	SE of kappa
	**+Ve** **(*n* = 16)**	**-Ve** **(*n* = 14)**						
** + Ve**	**4(a)**	**2(b)**	**0.250** **(0.319—to 0.651)**	**0.857** **(0.421—0.996)**	**0.667** **(0.185 -0.946)**	**0.500** **(0.377- 0.623)**	**0.103** **(0.279–0.484)**	**0.195**
** - Ve**	**12(c)**	**12(d)**						

^a^true positive, ^b^false positive, ^c^false negative, ^d^true negative

**Fig. 1:**
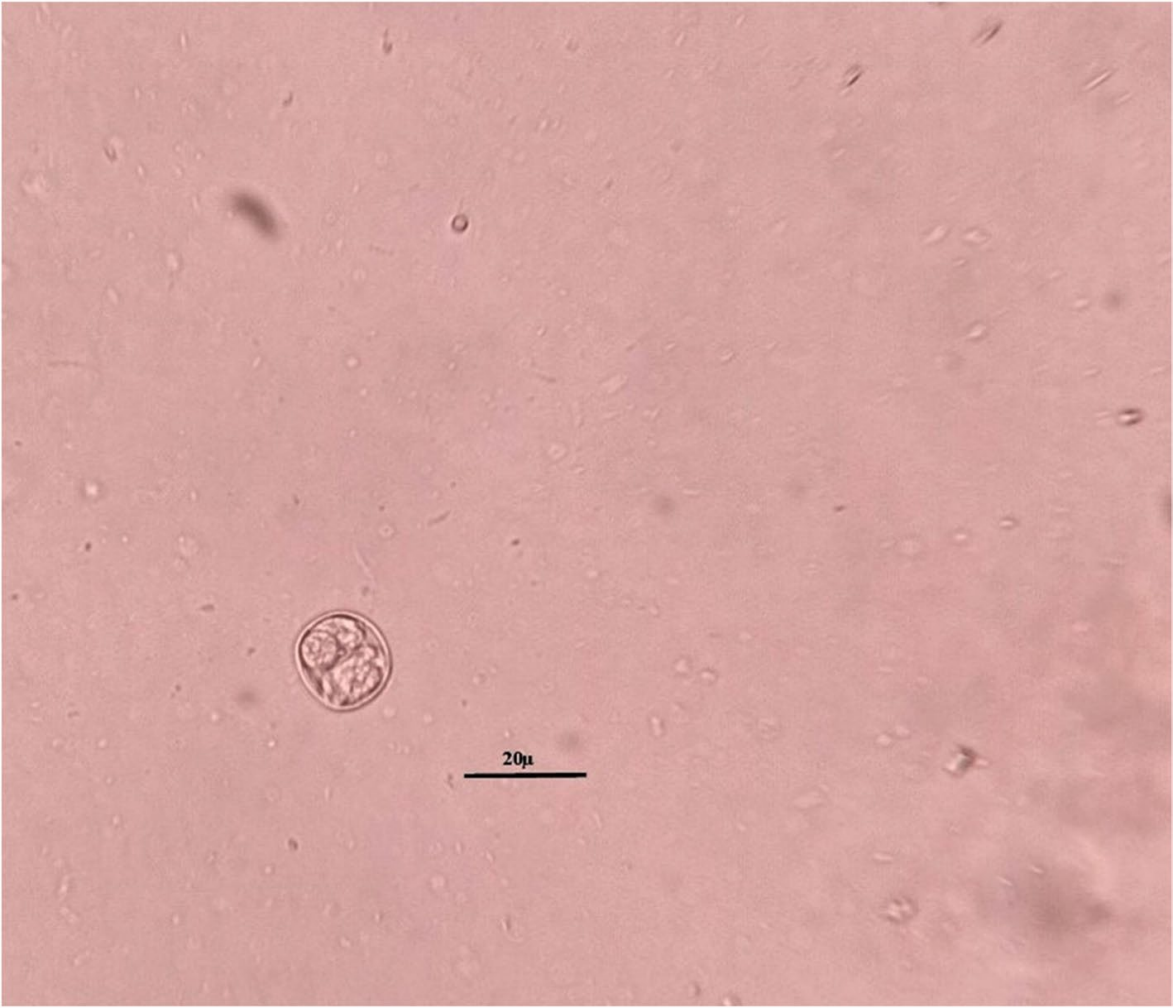
*S. tenella* sporocyst

Morphologically, the recovered sporocysts were oval in shape and measured 13.2–16.0 × 9.4–11 μm (Fig. 1).

### Molecular and phylogenetic description

**Fig. 2:**
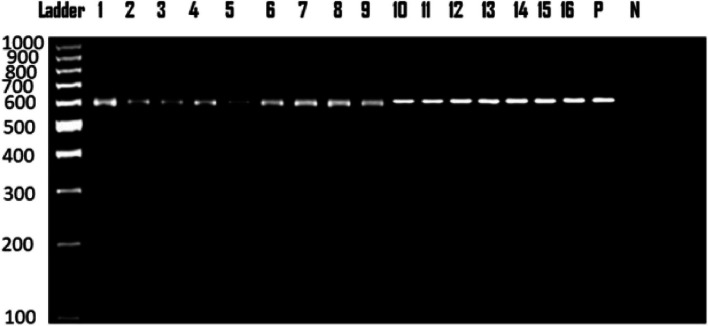
Gel electrophoresis of PCR product of 18S rRNA gene showing bands detected at 600 bp region belonged *S. tenella* L, DNA ladder (1kb); Lanes 1–16, fecal samples; P, Positive control; N, negative control

Sixteen out of the 30 PCR-tested samples yielded homogenous electrophoretic bands of 600 bp (Fig. 2).

**Fig. 3:**
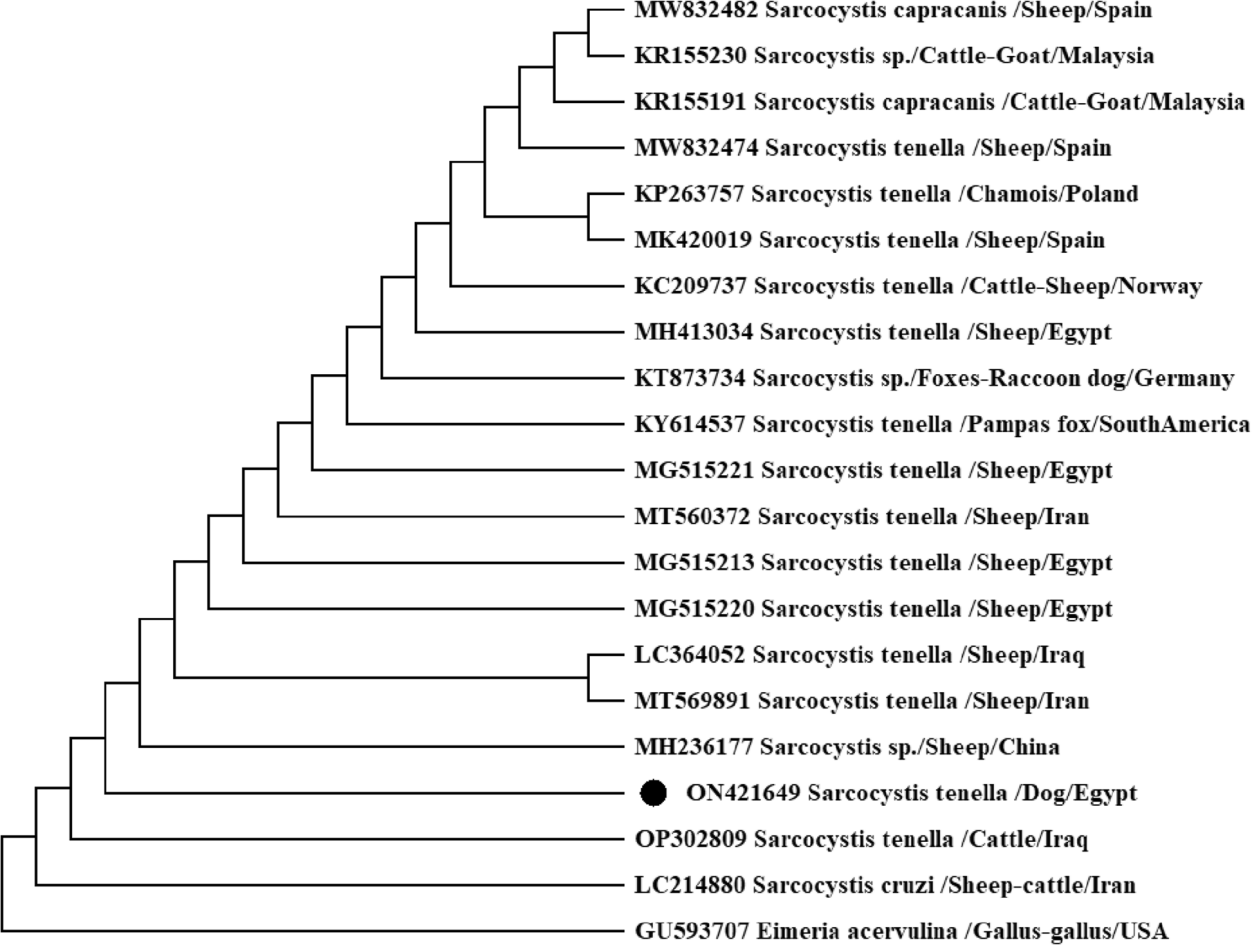
Phylogenetic tree of selected members of the family Sarcocystidae based on 18S rDNA sequences with *Eimeria acervulina* as the out-group, inferred using the Maximum Likelihood Tree implemented in MEGA software version 7. The GenBank accession numbers of all the sequences included in the analysis are given before the taxon names. The black circle identifies the sequence obtained in this study (ON421649)

Based on 18S rRNA nucleotide analysis, all isolates yielded identical nucleotide sequence (ON421649), which shared the highest similarity (99.82%) with those of *S. tenella* from sheep (MT569891, LC364052, OP302809 and MH236177) and 99.64% with cattle isolate, *S. cruzi* (LC214880). Although our isolate had 99.44% and 99.10% identity to *S. tenella* from Egyptian sheep (MG515213, MG515220, MG515221) and (MH413034), respectively. Furthermore, there was inter-specific nucleotide polymorphism with the aforementioned sequences which represented by one nucleotide deletion at 81/566 position. Also, comparison of our isolate with *S. tenella* from Pampas fox isolate (KY614537) revealed 99.15% nucleotide similarity. Moreover, 98.92% identity with the sequences of *S. capracanis* from goats and sheep, was also observed (MW832482) as depicted in Fig. 3.

## Discussion

*Sarcocystis* species represent an important member of the cyst-forming coccidian parasites. They involve several species with differences in their life cycle and pathological impact. Furthermore, scarce reports are published on the prevalence and species diversity of *Sarcocystis* in Egyptian dogs, and this study fills a knowledge gap in the epidemiology of animal sarcocystosis in Egypt.

The distribution pattern of *Sarcocystis* infection among screened dogs in this survey was nearly consistent with the previous record of Nathalia et al. [21] who investigated that the prevalence of *Sarcocystis* species from the intestinal scrapings and fecal samples of Pampas fox was 17.6%. Likewise, the estimate prevalence of dog sarcocystosis varies from 2.2 to 9% according to the worldwide reports [22–24]. Also, Abbas et al., [25] found that *Sarcocystis* oocysts were detected in 29 fecal samples from 1126 dogs with a very low pooled prevalence (2.0%) in Egypt, while El Menyawe and Abdel Rahman [26], and Sabry and Lotfy [27], depicted that the overall prevalence of dog sarcocystosis in Cairo and Giza governorates was 1.8% and 14.8%, respectively. In a recent study, a very low prevalence (0.3%) was recorded in dogs from Calgary, Canada [28]. However, some studies ranked a higher occurrence percentage of *Sarcocystis* infection (42–72% and 28.5%) in sheepdogs and domestic dogs from Perú and Ethiopia, respectively [29, 30]. These marked differences might be related to several factors such as the location, ownership status, sampling procedures, demographics, anthelmintic usage, and sensitivity of diagnostic techniques. Also, Egyptian dogs are frequently exposed to tissues of infected animals, particularly around slaughter houses with a free access to visceral organs, aborted fetuses and placenta of herbivorous animals [31]. Therefore, these dogs may be at the risk of contracting infections with cyst-forming parasites such as *Neospora* spp. and *Sarcocystis* spp. [32].

In relation to age, this survey disclosed that the prevalence of *Sarcocystis* spp. was age-dependent, with the highest infection rates in old age category (> 2 years). Similarly, Katagiri, and Oliveira-Sequeira [33] also endorsed a high prevalence of sarcocystosis in older dogs. This could be as a result of using older dogs for guarding purposes, thereby increasing their tendency to move around more frequently and possibly contract the infection, which elucidate the current findings. Moreover, puppies are less likely to hunt and therefore would probably be less prone to various sources of contamination. Contrary, the results of earlier studies indicated that puppies and young animals are more likely to be infected with helminths and protozoa than older dogs [34–36]. Also, our result was not in accordance with the findings of Adejinmi and Osayomi, Mirzaei, Awadallah et al. and Symeonidou et al. [24, 37–39] who reported the highest peak of protozoan parasites was found in younger dogs, as young puppies are supposed to various parasitic infections including *Sarcocystis* due to undeveloped immunity as the consequence of diminished level of passive immunity received from their dams [40].

In the current survey, the dog gender had no effect on *Sarcocystis* infection; although the prevalence was higher in male dogs as compared to females. The present figure disagrees with the finding of Zelalem and Mekonnen [41], who revealed a higher prevalence of *Sarcocystis* in the female dogs than males, and reasoned that due to the peculiar reproductive activities in the female animals may lead to stress and subsequent reduction in their immunity.

The current knowledge revealed that the utilize of light microscopy alone for broad and routine parasitological investigation of *Sarcocystis* infection has inadequate diagnostic significance. However, the molecular approaches could be the best tool to categorize and classify the *Sarcocystis* species; still, both microscopy and molecular analysis are vital and should be incorporated together for screening platforms [42–44]. Currently, the morphometric evidence of the recovered sporocysts of *S. tenella* was in line with the finding of Saito et al. [45], but it differs from those of red deer dog origin (*S. gracilis*, 15 × 10 μm) [2], and of sheep cat origin (*S. gigantea*, 10.5–14 × 8–9.7 μm and *S. medusiformis*, 10.3–13 × 7.3–8.8 μm [45]. Also, it morphometrically differs from the sporocysts of *S. arieticanis* of sheep dog origin (15–16 × 9–10 μm) [45]. Since no species has been found that parasitizes more than one genus of intermediate host according to investigations reported by Levine [46].

This is the first molecular evidence of the final host role of domestic dogs in the life cycle of *S. tenella* in Egypt. Prior to this study, to the best of our knowledge, no experimental evidence had been issued in support of this in Egypt, although sequences closely related to that of *S. tenella* were reported from living carnivores, the pampas fox (*Lycalopex gymnocercus*) sampled in South America (99.15% identity). Hence, the existence and validation of *S. tenella* in dog fecal sample emphasize the occurrence of the species in Upper Egypt. The current results may suggest that dogs analyzed in our study had fed on tissues that originated from small domestic ruminants, or that *S. tenella* or related *Sarcocystis* spp. had parasitized the prey of these dogs.

There are a few worldwide records on carnivores as definitive host (DH) for *Sarcocystis* spp. using fecal molecular and phenotypic analysis. In the study of Nathalia et al. [21] established that Pampas fox (*Lycalopex gymnocercus*) was proposed as definitive host for *S. cruzi*, *S. tenella* and possibly various *Sarcocystis* spp. using birds as intermediate hosts (IH). Also, More et al*.* [13] found that the most often recognized *Sarcocystis* spp. in the mucosal scraping of fox, were *S. tenella* or *S. capracanis* (10.0%); *S. miescheriana* (8.0%) and *S. gracilis* (8.0%) followed by *Sarcocystis* spp., which use birds as intermediate hosts (6.0%), and *S. capreolicanis* (4.0%), however in the raccoon dog samples, sequences with a ≥ 99% identity with the following species were detected: *S. miescheriana* (18.4%), *S. gracilis* (13.1%), *Sarcocystis* spp. using birds as IH (10.5%), *S. tenella* or *S. capracanis* (2.6%) and *S. capreolicanis* (2.6%). In another DNA sequence-based revision in Hungary, the recovered isolate from dog fecal samples was identified based on 100% identity with already reported sequences of *S. morae* from cervids in Lithuania and Spain [47]. On the other hand, *S. tenella* isolates (MG515213, MG515220515221) and (MH413034) identified in sheep from Egypt by Elmishmishy et al. [48] and El-Morsey et al. [49] shared an interspecific identity of 99.44% and 99.10%, respectively with *S. tenella* isolate (ON421649) detected herein.

## Conclusions

Our survey presents a molecular scheme to categorize *Sarcocystis* spp. infections in the fecal specimens from dogs collected in Aswan province, Egypt. The results show that a method comprising of 18S rRNA gene amplification, cloning and sequencing is suitable to ascertain *Sarcocystis* spp. infections and to identify potential DH of these parasites. Further revisions aimed at recognizing the complete life-cycle, and the pathological impact of these infections on DH health remains necessary. Additionally, surveys for monitoring the epidemiological and taxonomic status of *Sarcocystis* infections using multiple marker genes in Egyptian livestock and carnivore populations should be highlighted.

### Supplementary Information

**Additional file 1.** Ph. ( ): Specific genomic product for Sarcocystis sample with ≈ 600 bp. Ph. ( ): Computerized detection Specific genomic product for Sarcocystis sample with ≈ 600 bp. Ph. ( ): Computerized fragment length calculation of specific genomic product for Sarcocystis sample with ≈ 600 bp.

## Data Availability

The sequence identified in the study is available in the public domain database of NCBI under Accession No. ON421649. https://www.ncbi.nlm.nih.gov/nuccore/ON421649
