# Supplementary material for: Prevalence and first molecular identification of Sarcocystis species in feces of domestic dogs (Canis familiaris) in Egypt
Source: BMC Vet Res. 2023 Dec 18;19:278. doi: 10.1186/s12917-023-03841-8 (PMC10726512; doi:10.1186/s12917-023-03841-8)
Supplement: Supplementary file 1 — Additional file 1. Ph. ( ): Specific genomic product for Sarcocystis sample with ≈ 600 bp. Ph. ( ): Computerized detection Specific genomic product for Sarcocystis sample with ≈ 600 bp. Ph. ( ): Computerized fragment length calculation of specific genomic product for Sarcocystis sample with ≈ 600 bp. [file 12917_2023_3841_MOESM1_ESM.docx]

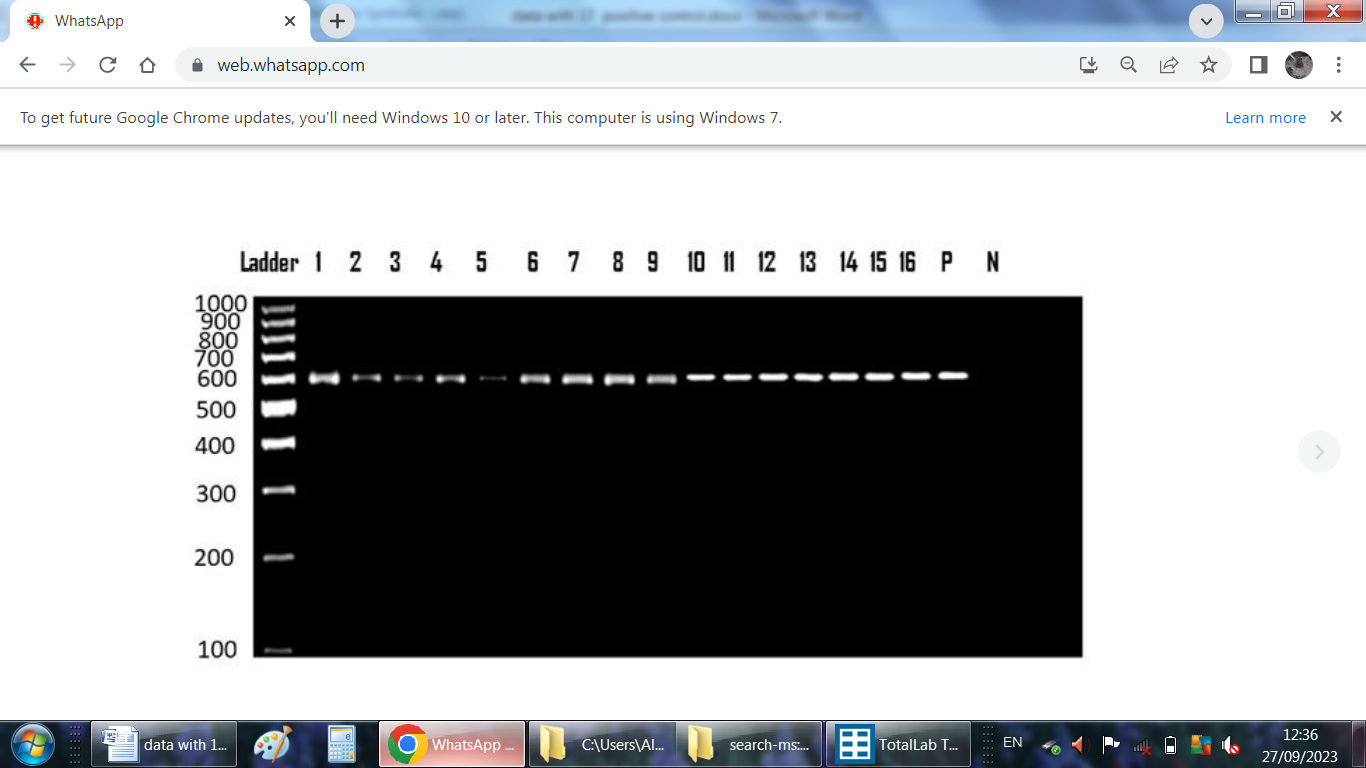


# Ph. ( ): Specific genomic product for Sarcocystis sample with ≈ 600 bp.


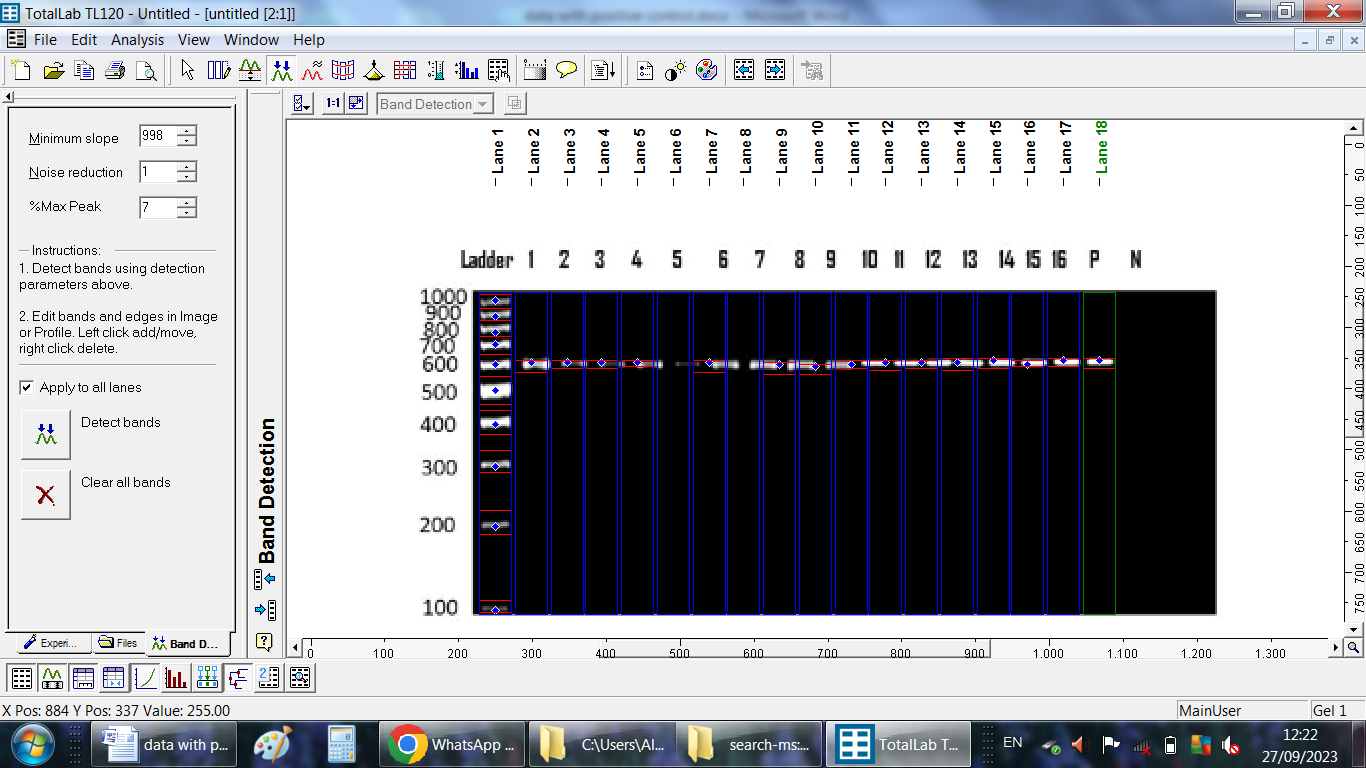


# Ph. ( ): Computerized detection Specific genomic product for Sarcocystis sample with ≈ 600 bp.


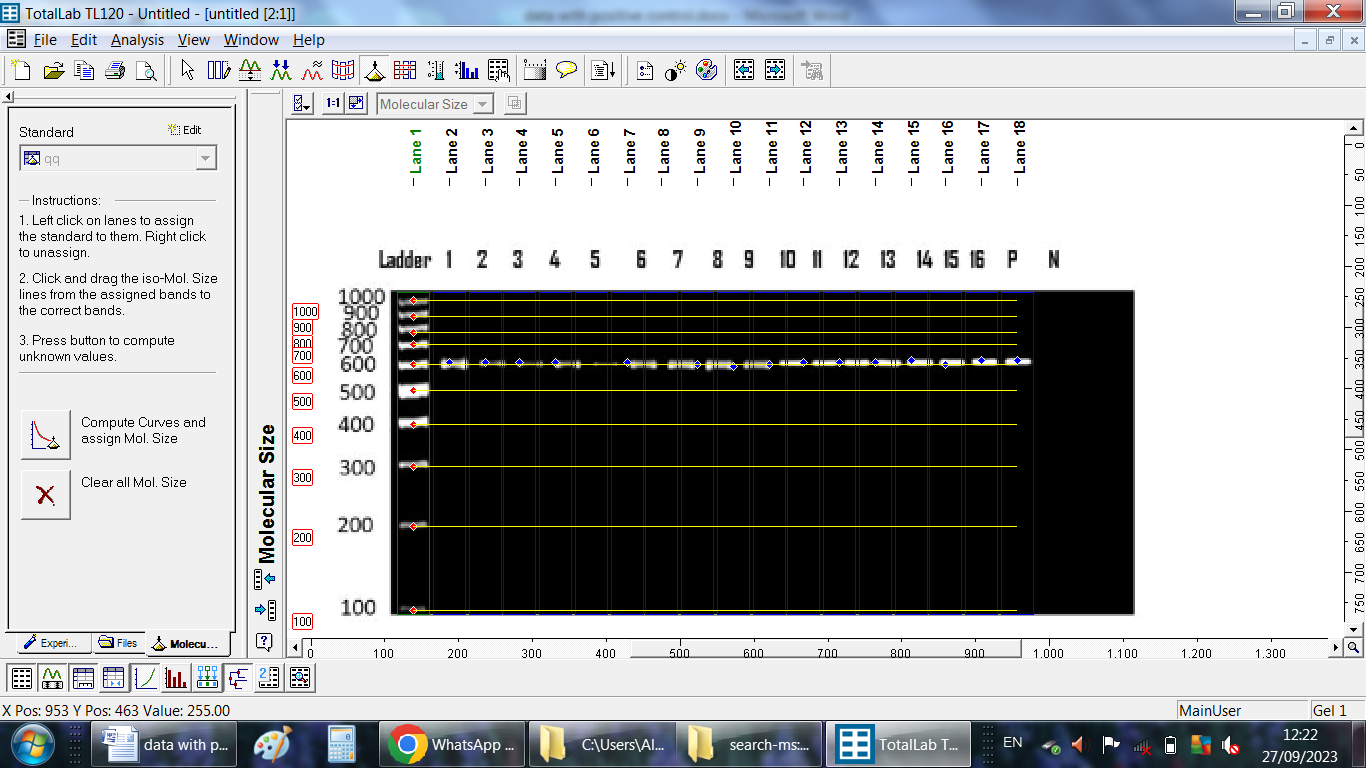


# Ph. ( ): Computerized fragment length calculation of specific genomic product for Sarcocystis sample with ≈ 600 bp.
